# A study of the effects of therapeutic doses of ionizing radiation in vitro on *Lactobacillus* isolates originating from the vagina - a pilot study

**DOI:** 10.1186/s12866-016-0716-5

**Published:** 2016-05-31

**Authors:** Tomasz Gosiewski, Tomasz Mróz, Dorota Ochońska, Wojciech Pabian, Malgorzata Bulanda, Monika Brzychczy-Wloch

**Affiliations:** Department of Bacteriology, Microbial Ecology and Parasitology, Chair of Microbiology, Jagiellonian University Medical College, 18 Czysta Street, 31-121 Krakow, Poland; Institute of Biology, Pedagogical University, 2 Podchorazych Str, 30-084 Krakow, Poland; Department of Infection Epidemiology, Chair of Microbiology, Jagiellonian University Medical College, 18 Czysta Str, Krakow, Poland; Department of Gynaecology and Endocrinology, University Hospital, 23 Kopernika Str., 31-501 Krakow, Poland

**Keywords:** Ionizing radiation, Microbial flora, *Lactobacillus*

## Abstract

**Background:**

Ionizing radiation is used as a therapeutic option in the treatment of certain neoplastic lesions located, among others, in the pelvic region. The therapeutic doses of radiation employed often result in adverse effects manifesting themselves primarily in the form of genital tract infections in patients or diarrhea. The data available in the literature indicate disorders in the microbial ecosystem caused by ionizing radiation, which leads to the problems mentioned above. In the present study, we examined the influence of ionizing radiation on 52 selected strains of bacteria: *Lactobacillus crispatus, L. fermentum, L. plantarum, L. reuteri, L. acidophilus L. amylovorus, L. casei, L. helveticus, L. paracasei, L. rhamnosus, L. salivarius* and *L. gasseri*. This collection of *Lactobacillus* bacteria isolates of various species, obtained from the genital tract and gastrointestinal tract of healthy women, was tested for resistance to therapeutic doses of ionizing radiation.

**Results:**

The species studied, were isolated from the genital tract (*n* = 30) and from the anus (*n* = 22) of healthy pregnant women. Three doses of 3 Gy (fractionated dose) and 50 Gy (total dose of the whole radiotherapy cycle) were applied.

The greatest differences in survival of the tested strains in comparison to the control group (not subjected to radiation) were observed at the dose of 50 Gy. However, the results were not statistically significant. Survival decrease to zero was not demonstrated for any of the tested strains.

**Conclusions:**

Therapeutic doses of radiation do not affect the *Lactobacillus* bacteria significantly.

## Background

In 1895, Wilhelm Roentgen discovered X-rays, and as soon as a year later, in 1896, Émil Grubbé made the first attempt to employ them in the treatment of breast cancer [1, 2]. In 1897, the first report appeared describing intestinal damage resulting from the use of X-rays in radiotherapy [3], while in 1900 Paul Ulrich Vilard discovered gamma rays [4]. Currently, radiotherapy utilizing gamma rays, and charged particle beams, is permanently present in oncology as a treatment method for certain types of cancer located, among others, in the pelvis.

Ionizing radiation, apart from the desired effect, that is damaging or destruction of cancer cells, also affects the surrounding normal tissues. With respect to gamma rays, the interaction with the molecules and cell structures takes place in an indirect manner [5]. Under the influence of gamma rays, radiolysis of water occurs with the formation of reactive oxygen species containing an unpaired electron in the valence shell. Reactive oxygen species (ROS) are very reactive because of that and cause tissue damage through the damage to the DNA chain and cellular structures [6]. Because the intestinal microbiota comprises mainly anaerobic bacteria, ROS is affected by extremely destructive to their cells. Intestinal ecosystem disorders may influence on the overall condition of the digestive system. In the event of irradiation of the abdomen or pelvis, the accompanying clinical symptom may be the appearance of diarrhea [1, 7, 8] and infections in the female genital tract [9].

Both the intestines and the vagina are colonized by bacteria in an abundant way, which significantly cooperate with the host organism to retain its state of homeostasis. The commensal microbiota of the gastrointestinal tract plays an important role in the process of digestion and absorption of nutrients as well as it displays the protection against the invasion of pathogenic microbes by establishing the resistance to their colonisation and influencing the host immune system [10]. The number of bacterial cells inhabiting the human gastrointestinal tract is growing considering both qualitative and quantitative aspects starting from the stomach and ending at the colon. In the stomach there are very few bacteria due to the low pH of the gastric juice and these are the species which got there with the digestive tract content and remain there for a very short period of time. In the duodenum and the jejunum the number of bacterial cells increases and equals approximately 10^3^–10^4^ of bacterial cells/ml (cfu/ml)-these are mostly representative species of *Streptococcus* and *Lactobacillus* [11]. In the ileum the number of bacterial cells equals 10^7^–10^8^ cfu/ml and there is an increase in species diversity [11]. In the whole large intestine the bacterial population equals about 10^14^ (i.e., 100 trillion)-in 1 ml of the digestive tract content the number of the bacterial cells amounts approximately 10^11^ cfu/ml [12]. The colonic microflora is primarily composed of anaerobic bacteria (*Clostridium, Eubacterium, Bacteroides, Bifidobacterium*) and in smaller numbers, representatives of aerobic and relatively anaerobic bacteria (*Enterobacteriaceae*, *Lactobacillus, Streptococcus, Staphylococcus,*) [13]. The vagina is dominated largely by bacteria of the genus *Lactobacillus* and, in smaller numbers, *Bacteroides*, *Bifidobacterium* or *Escherichia coli* and *Streptococcus* [14]. The role of the intestinal flora has been repeatedly confirmed in the course of many diseases, such as chronic inflammatory bowel disease (IBD) [15, 16], irritable bowel syndrome (IBS) [17], allergies [18] or even metabolic diseases [19]. By analogy, it has been demonstrated that disorders of the vaginal flora contributed to the emergence of infections in the reproductive tract. This concerns mainly the reduction of the population of bacteria of the genus *Lactobacillus* [20, 21].

For many years, research has been carried out on probiotics, namely microorganisms (mostly bacterial) exerting beneficial effects on the host organism. Probiotics primarily include bacteria of the genus *Lactobacillus*. A question arises if, among this significant group of bacteria, there are strains with varying resistance to radiation at therapeutic doses applied in patients with tumors of the pelvis and abdomen. This is an important issue as it is necessary to minimize the negative side effects of radiation therapy and improve the quality of life for patients. In order to do this, a collection of *Lactobacillus* bacteria isolates of various species, obtained from the genital tract and gastrointestinal tract of healthy women, was tested for resistance to therapeutic doses of ionizing radiation, equivalent to a fractionated dose of 3 Gy and a total dose of radiotherapy throughout the cycle of 50 Gy, which are used in external beam radiotherapy for cancer.

## Results

**Fig. 1:**
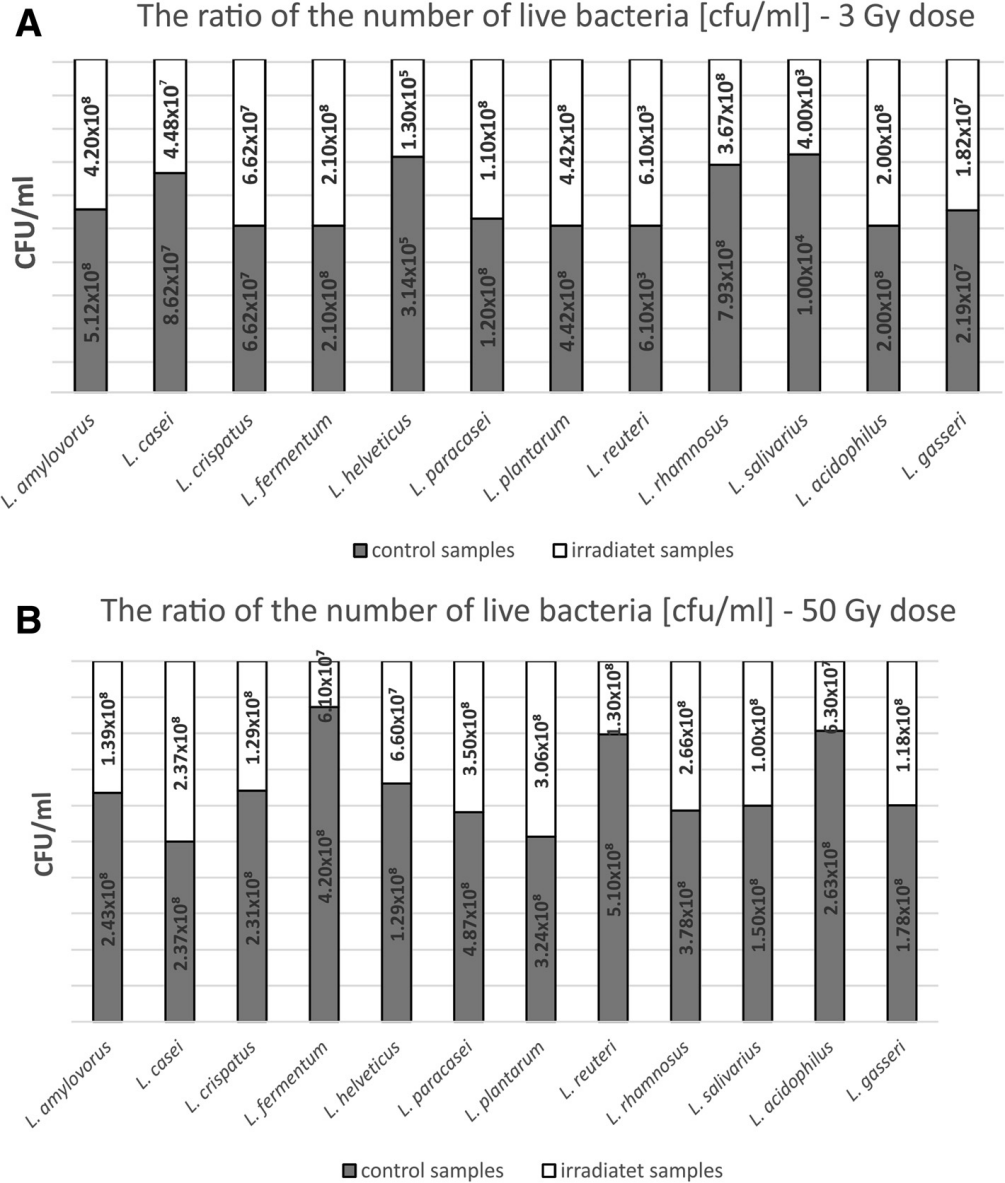
Average survival of strains within the test species *Lactobacillus* subjected to ionizing radiation as compared to control: **a** 3 Gy dose; **b** 50 Gy dose

The collection of 52 bacterial isolates of the species *Lactobacillus* were subjected to irradiation at doses of 3 Gy and 50 Gy. The species studied, i.e., *L. amylovorus, L. casei, L. helveticus, L. paracasei, L. rhamnosus, L. salivarius* and *L. gasseri*, exhibited a decrease in viability compared to the control samples; however, the results were not statistically significant (Fig. 1a). No differences between the test samples and controls in the average survival of strains belonging to the following bacterial species: *L. crispatus, L. fermentum, L. plantarum, L. reuteri* and *L. acidophilus* for the radiation dose of 3 Gy were showed. When the total dose of 50 Gy was applied, no change was demonstrated in viability of strains belonging to two species: *L. casei* and *L. plantarum*; in the remaining cases, bacterial viability decreased compared to control samples, but the differences were not statistically significant (Fig. 1b). A drop in the survival rate to zero was not found for any of the test strains regardless of the dose of ionizing radiation applied.

## Discussion

Radiation therapy of neoplastic lesions located in the pelvis is a commonly used therapeutic method. Ionizing radiation has an effect not only on tumor cells but also affects normal tissues which is associated with certain symptoms in patients. Radiation to the pelvic area is most frequently associated with the occurrence of chronic diarrhea, which is observed in as many as 80 % of patients [22, 23]. Also, vaginal flora is altered in women undergoing radiation therapy, observed through a significant preponderance of anaerobic bacterial species in these patients. Meanwhile, the composition of aerobic species does not show significant changes in comparison with healthy women [9, 24].

In order to improve the quality of life for patients, the objective is to mitigate the frequency of bowel movements through the application of supplementation containing probiotic bacterial strains. Demers et al. conducted research on 229 patients after irradiation of the pelvis with a formulation comprising *Lactobacillus acidophilus* LAC-361 and *Bifidobacterium longum* BB-536 and concluded that, after 60 days of treatment with the preparation, the frequency of defecation in the tested patients was reduced as compared to placebo [22]. Similar observations were made by Urbancsek et al., and Chitapanarux et al., who showed that patients supplemented with the preparation containing *Lactobacillus* and *Bifidobacterium* had significantly fewer bowel movements than patients receiving placebo [23, 25]. The literature lacks descriptions of attempts to employ probiotics in the treatment of disorders of the vaginal flora in women undergoing radiation therapy. The quoted above results of the study prompted us to attempt to select strains of probiotic bacteria with increased resistance to ionizing radiation at doses applied in classical radiotherapy of the pelvic area which was the aim of this study. The test strains were obtained from the genital tract of healthy pregnant women and were all classified to the genus *Lactobacillus*. The two doses of ionizing radiation applied (3 Gy-fractionated dose) and 50 Gy (total dose for the full cycle of radiotherapy) did not reduce the survival of the studied strains in a statistically significant way, although the dose of 50 Gy was more effective. The results obtained suggest that therapeutic doses of ionizing radiation do not significantly affect the survival of bacteria of the genus *Lactobacillus* living in the gastrointestinal tract or the reproductive tract of patients undergoing radiation therapy. Hwanga et al. demonstrated that bacteria from the genus *Lactobacillus* reduced their viability to zero at radiation doses of at least 1000 Gy and higher [26]. Despite this, patients often report difficulty resulting from, mainly, the occurrence of diarrhea. Possibly, ionizing radiation damages the intestinal epithelium, which enables direct contact of the intestinal flora with the immune system of the gastrointestinal tract, which, in turn, leads to the development of local inflammation and diarrhea [27, 28]. Inflammation of the gastrointestinal tract consequently induces a change in the quantitative composition of the individual components of the intestinal flora, which disrupts the entire intestinal ecosystem and intensifies gastrointestinal symptoms, which was repeatedly reported by researchers [12]. If, however, supplementation with probiotic bacteria applied for patients undergoing radiation therapy brings effects in the form of a reduction in the number of diarrhea cases [22, 23], then, it may be concluded that there is no need to look for a radioresistant probiotic strain and it is enough to apply the already familiar strains, which are already much more resistant to ionizing radiation than eukaryotic cells.

## Conclusions

Therapeutic doses of radiation do not affect the *Lactobacillus* bacteria significantly, therefore, the described disorders in the composition of the bacterial flora in irradiated patients may be the result of changes in the host environment of commensal bacteria.

## Methods

### Lactobacillus isolates

The 52 isolates examined came from a collection gathered throughout the year 2010 at the Chair of Microbiology of the Jagiellonian University Medical College and derived from routine microbiological diagnostics of the female genital tract (*n* = 30) and the anus (*n* = 22) in healthy pregnant women., carried out in our microbiology laboratory.

Inclusion criteria: women between the ages of 18 and 40; pregnant women in the first trimester of gestation (1st–13th week of gestation); without clinical symptoms of genitourinary infections, requiring the application of antibiotherapy; confirmed physiological composition of genitourinary tract flora according to the 10-point Nugent score (results: 0–6 value); written permission to take part in the study.

Exclusion criteria: women under 18 and over 40; pregnant women with the so-called high-risk pregnancy; rupture of membranes; gestational diabetes; application of antibiotherapy in the period of up to 30 days before getting pregnant or during gestation; diagnosis of bacterial vaginosis, diagnosed on the basis of a direct smear from a vaginal swab stained with the use of Gram’s method (results 7–10 points in the 10-point Nugent score) and on the basis of culture; clinical symptoms of genitourinary infection requiring antibiotherapy; lack of a written permission to take part in the study.

The study was approved by Jagiellonian University Bioethical Committee decisions No. KBET/47/B/2009.

Each isolate has been identified to species level by sequencing 16S rRNA [29]: *L. amylovorus* (*n* = 5)*; L. casei* (*n* = 3)*; L. crispatus* (*n* = 10)*; L. fermentum* (*n* = 3)*; L. helveticus* (*n* = 3)*; L. paracasei* (*n* = 3)*; L. plantarum* (*n* = 5)*; L. reuteri* (*n* = 3)*; L. rhamnosus* (*n* = 4)*; L. salivarius* (*n* = 3)*; L. acidophilus* (*n* = 3)*; L. gasseri* (*n* = 7).

### Reference strains

A few reference strains of the genus *Lactobacillus* were used in the research: *L. fermentum* (ATCC 20052); *L. plantarum* (ATCC 20174); *L. plantarum* (ATCC 14431); *L. acidophilus* (ATCC 4356).

### Bacterial cultures

The tested strains were cultured in anaerobic conditions at 37 °C for 48 h on de Man-Rogosa-Sharpe MRS agar (Oxoid).

### Preparing strains for irradiation

A 4-ml suspension of 5 McFarland (1,5x10^9^ CFU/ml) density was prepared in normal saline immediately before radiation. It was divided into a 2-ml test group (subjected to radiation) and a 2-ml control. The samples were placed into plastic Eppendorf tubes.

### Irradiation of strains

The test samples placed in a cobalt collimator were irradiated at doses of 3 Gy and 50 Gy, corresponding to the fractionated and total doses (doses added up over the complete cycle of radiotherapy) used in external beam radiotherapy for cancer. To irradiate the samples, a Theratron 780E unit was used containing ^60^Co source with an activity of 42.8 TBq for a day of irradiation emitting quanta of gamma rays of the energy of 1173.237 keV (99 %) and 1332.501 keV (99 %), provided by the Department of Radiation Physics and Dosimetry at the Henryk Niewodniczanski Institute of Nuclear Physics of the Polish Academy of Sciences in Kraków. Control samples underwent exactly the same procedure as the test samples, except for undergoing radiotherapy.

### Strains viability

The test and control samples were serially diluted in MRS broth (Oxoid) and plated onto the MRS Agar (Oxoid). Afterwards, plates were cultured in anaerobic conditions at 37 °C for 48 h. The cultivated colonies were counted to determine the number of cells in 1 ml (CFU/ml).

### Statistical analysis

The Mann-Whitney *U* test was used for analysis of differences between the irradiated samples and control samples within each species. The value *p* < 0.05 was regarded as the threshold for statistical significance. All analyses were conducted using SAS 9.1 package and SAS Enterprise Quide 3.0 (SAS Institute, USA).

## Abbreviations

ATCC, American type culture collection; cfu, colony forming unit; Gy, the gray is a derived unit of ionizing radiation dose in the International System of Units (SI); IBD, inflammatory bowel disease; keV, kiloelectron-volts; ROS, reactive oxygen species; TBq, tertabecquerel the SI derived unit of radioactivity
